# Supporting change in a complex reality: a qualitative study of specialist nurses’ perceptions of counselling patients at risk of type 2 diabetes

**DOI:** 10.1016/j.ijnsa.2026.100607

**Published:** 2026-06-24

**Authors:** Åsa Carlsund, Åsa Hörnsten, Fatima Hoosen, Stephen Amoah, Julia Otten

**Affiliations:** a Department of Nursing, Umeå University, Umeå, Sweden; b Biomedical Research and Innovation Platform, South African Medical Research Council, Cape Town, South Africa; c Health through Physical Activity, Lifestyle and Sport Research Centre (HPALS), Division of Physiological Sciences, Department of Human Biology, University of Cape Town, Cape Town, South Africa; d Transdisciplinary Research Area "Technology and Innovation for Sustainable Futures" and Center for Development Research (ZEF), University of Bonn, Bonn, Germany; e Department of Public Health and Clinical Medicine, Umeå University, Umeå, Sweden

**Keywords:** Cultural, Dietary advice, Gender, Prediabetes, Risk, Specialist nurse, Type 2 diabetes

## Abstract

**Background:**

Specialist nurses in primary health care play a key role in supporting individuals at risk of type 2 diabetes to adopt healthier eating habits. Although dietary counselling is central to prevention, limited knowledge exists on how nurses experience providing individualized advice that considers gender, cultural background, and patients’ everyday contexts.

**Objective:**

To explore specialist nurses’ perceptions of delivering individualized dietary advice to patients at risk of type 2 diabetes, with attention to gender, culture, and contextual factors.

**Design:**

Qualitative study based on semi-structured interviews.

**Setting and participants:**

Specialist nurses in Swedish primary health care were recruited through snowball sampling. Data were collected between August and November 2025.

**Methods:**

Data were analysed using qualitative content analysis according to Graneheim and Lundman.

**Results:**

Four themes and 12 subthemes described how specialist nurses applied patient-centered strategies, considered gendered and multicultural influences, encountered clinical and motivational challenges, and expressed a need for enhanced organizational and educational support. Nurses emphasized empathy, small-step goal setting, and person-centered communication. However, limited consultation time, low patient motivation, socioeconomic constraints, and cultural complexity hindered effective counselling. Participants highlighted the need for cultural competence training, accessible and updated resources, and stronger organizational support.

**Conclusion:**

Effective dietary prevention requires that specialist nurses have sufficient time, appropriate tools, and organizational support to tailor advice to patients’ everyday lives. Despite strong commitment, structural, cultural, and motivational barriers limit the effectiveness of prevention efforts. Strengthening organizational structures, improving access to culturally adapted resources, and adopting collaborative care models may enhance diabetes prevention in clinical practice.

**What is already known**

• Specialist nurses play a crucial role in providing patient-centred dietary advice, but they encounter barriers like limited time and resources.
• Patients often see counselling as supportive but hard to turn into lasting everyday practices.

**What this paper adds**

• Specialist nurses emphasize personalized, step-by-step, and patient-centred approaches as crucial for delivering effective dietary advice.
• Organizational constraints, limited training, and the lack of structured support hinder effective type 2 diabetes prevention in practice.

Alt-text: Unlabelled box dummy alt text

## 1 Background

Prediabetes is a common metabolic condition associated with an elevated risk of developing type 2 diabetes and other cardiovascular diseases (Unnikrishnan et al., 2025). Studies emphasize ongoing gaps in implementing evidence-based preventive measures in routine healthcare, where many patients do not receive recommended screening, counselling, or follow-up (Echouffo-Tcheugui et al., 2025). This reveals a significant gap between established clinical knowledge and real-world outcomes (Echouffo-Tcheugui et al., 2025; Green et al., 2024; Tseng et al., 2023). Specialist nurses are expected to provide personalized, patient-centred dietary advice while considering behavioural, cultural, and socio-economic factors that influence lifestyle change. Their role also includes supporting patients’ motivation and tailoring recommendations to daily life, often within time- and resource-constrained settings (Echouffo-Tcheugui et al., 2025; Herman, 2023; Sandforth et al., 2025).

Lifestyle changes are the primary way to prevent type 2 diabetes in people with prediabetes, and structured interventions can lower risks, including dietary tweaks, increased physical activity, and behavioural support (Herman, 2023; Unnikrishnan et al., 2025). However, practical implementation remains limited. Specifically, participant recruitment is low, compliance is poor, and structural barriers such as costs and limited access to effective programs hinder progress at the population level (Echouffo-Tcheugui et al., 2025).

Research also shows that patients consider that lifestyle consultation with specialist nurses is essential, but often insufficient. They want more time, clear and personalized advice, and further explanations of laboratory values and risks, not only statements that they are high or low. Many feel that the support is well-intentioned but sometimes hard to put into practice in daily life (Hills et al., 2025; Serrano-Ripoll et al., 2025). Digital solutions are appreciated especially when they deliver short, simple messages and supplement face-to-face meetings (Serrano-Ripoll et al., 2025). Patients report that motivation, in particular, increases when advice is tailored to their life situation (Matrook et al., 2024).

Specialist nurses, on the other hand, consider lifestyle counselling a key element in supporting self-management among people at risk of or with type 2 diabetes, but they face several challenges, such as limited time, unclear role boundaries, and insufficient training in behavioural change (Pot et al., 2025). They also request structured tools, access to digital support, and organizational space for longer consultations (Pot et al., 2025). Studies further emphasize that family-based models enhance counselling effectiveness but require additional resources and expertise (Hills et al., 2025; Serrano-Ripoll et al., 2025). Specialist nurses highlight the importance of incorporating lifestyle discussions into both primary and secondary care, focusing on person-centred communication and continuity. Digital interventions are viewed as promising, but they require training to be used effectively (Hills et al., 2025; Matrook et al., 2024; Pot et al., 2025; Serrano-Ripoll et al., 2025). Unfortunately, as several reviews have reported, even when nurse-led interventions are effective, inconsistencies in intervention design and a lack of standardized approaches reduce their overall impact (Pot et al., 2025; Tantayotai et al., 2023). Despite this growing body of research, there is a lack of in-depth understanding of how specialist nurses themselves experience delivering tailored dietary advice for diabetes prevention, particularly in relation to cultural, gendered, motivational, and organizational challenges. To understand why the impact is low, we wanted to explore perceptions from diabetes specialist nurses.

### 1.1 Objective

The study aimed to describe perceptions of specialist nurses in providing tailored dietary advice to patients at risk of type 2 diabetes, taking into account their whole life situation, including cultural and gender aspects.

### 1.2 Design and setting

This study used a qualitative approach, presenting findings from analysis of interviews with specialist nurses working as diabetes specialist nurses and district nurses in primary health care in Sweden. Data was analysed through qualitative content analysis. (Graneheim and Lundman, 2004; Lindgren et al., 2020).

### 1.3 Participants

The participants' (n = 11) ages ranged from 34 to 60, with an average of 43.6 years. Nine were women and two men. All were specialist nurses in primary health care in the north of Sweden, including two diabetes specialist nurses, five district nurses (registered specialist nurses working autonomously in primary health care), and four had both diabetes specialist and district nurse education (Table 1).

**Table 1:** Descriptions of the participants (n = 11).

	
Age range (mean)	34–60 (43.6)
Sex Women Men	9 2
Specialist Nurse Education (n)	
Diabetes specialist nurse	2
District nurse	5
Both Diabetes specialist nurse and District nurse	4

## 2 Methods

### 2.1 Data collection

A snowball sampling method was used to recruit nurses for the study. The first person approached in the sampling process was a research nurse working within a regional healthcare authority, who then provided recommendations for three potential participants. After receiving both oral and written information about the study's aims and procedures and then giving informed consent to participate in interviews, they were asked for suggestions of other professionals who could offer similar or complementary perspectives. In this way, the sample gradually expanded through the participants' networks. The process was repeated until enough diverse and rich material was collected. Data collection occurred from August to November 2025. The interviews were conducted at the participants' workplaces, lasted between 28 and 44 min (with a mean of 35.1 min), and were recorded using Microsoft Teams. To capture contextual details that might otherwise be missed, an interview guide with background and open-ended questions was used. The guide included questions such as, “In general, what are your thoughts about recommending dietary advice to high-risk patients with the goal of preventing type 2 diabetes, How would you feel about giving specific dietary advice based on a person’s gender or cultural background, How easy or difficult do you view it to implement specific dietary advice to people at risk in order to prevent type 2 diabetes. Which resources or support would you expect to be needed if dietary advice should be better adapted to a person's gender, societal, or cultural background?” Data collection was stopped once data saturation was reached, meaning no new meaning units appeared from the data (Graneheim and Lundman, 2004; Lindgren et al., 2020).

### 2.2 Analysis

The interviews were transcribed verbatim in Swedish. Translation into English was performed by the researchers (ÅC, ÅH), who are fluent in both Swedish and English, to ensure conceptual equivalence rather than literal translation. The text was then manually analysed using the qualitative content analysis method outlined by Graneheim and Lundman (2004) and Lindgren et al. (2020). The analysis began with a thorough reading to gain a general understanding of the material, which was then discussed in the research group. Thereafter, the continued analysis, now in the decontextualization phase, approached the text line by line, identifying meaning units that answered the aim. These were marked, then condensed to make them shorter while retaining the core. The condensed meaning units were then labelled with shorter codes. In the next phase, the recontextualization, the codes were compared and grouped based on similarities and differences. Through interpretation and abstraction, these were organized into preliminary sub-themes and themes, which were discussed and decided upon by the entire research group. The analytical process was dynamic and iterative, involving repeated movement between raw data and interpretative levels (Graneheim and Lundman, 2004; Lindgren et al., 2020).

### 2.3 Trustworthiness

To ensure trustworthiness, the study focused on credibility, dependability, and transferability. Qualitative content analysis was chosen as it offers a structured approach to describing variations in experiences (Graneheim and Lundman, 2004; Lindgren et al., 2020). Decontextualization and recontextualization were used to systematically capture and organize the data while preserving participants’ meanings. Credibility was enhanced through the inclusion of representative quotations and disconfirming cases, as well as through repeated discussions among the research team until consensus was reached. Ethical approval was obtained from the Swedish Ethical Review Authority, and the study adhered to the principles of the Helsinki Declaration. Detailed descriptions of participants, data collection, and analysis support transferability, allowing readers to assess the relevance of the findings to other contexts.

### 2.4 Ethical considerations

The study adhered to the ethical standards of the World Medical Association and the Helsinki Declaration. Ethical approval was obtained from the Swedish Ethical Review Authority (Dno: 2025–00,193-01–69,890). Participation was entirely voluntary, and all participants gave written informed consent. Measures were taken to safeguard their integrity during the study. Each consent was associated with a unique participant number, which was preserved throughout all research stages.

## 3 Results

The analysis identified four themes and twelve subthemes that describe specialist nurses’ experiences in providing individualized dietary advice to patients at risk of type 2 diabetes. The themes illustrate how specialist nurses use patient-centered strategies, consider gendered and multicultural influences, face clinical and motivational challenges, and express a need for organizational and educational support. The themes and their corresponding subthemes are presented below (Table 2).

**Table 2:** Themes, and subthemes.

Themes	Subthemes
Focusing on patient goals and strategies	Use patient-centred nursing Take one step at a time Apply understanding and caring
Taking gender and cultural aspects into account	Gender specific elements Multicultural dimensions Family and social situation
Challenges associated with implementing changes to prevent type 2 diabetes	Patient lack of motivation Implementation difficulties in daily life Clinical time constraints
Requesting additional support and resources	Cultural competence education Easy access to new knowledge Organizational backing

### 3.1 Focusing on patient goals and strategies

The specialist nurses used various strategies, including patient-centred nursing, taking one step at a time, and applying understanding and caring during the patient encounter.

### 3.2 Use patient-centred nursing

A person-centred nursing approach was utilized during patient meetings, where specialist nurses actively listened, reflected on patients' statements, and customized their advice to individual needs and circumstances. By consistently working to establish a trusting relationship and exploring the patient's life situation, the foundation was set for a deeper understanding of health behaviours and motivations for change. Motivational interviewing (MI) was used to support the patient's own motivation and decision-making.

*Motivational interviewing is a valuable tool because it doesn't involve pointing fingers. It focuses on helping the person to come to their own decisions*. (IW1)

The interaction was characterized by confirmation and respect, with an emphasis on encouraging autonomy and respect, without pointing out mistakes, aligning with evidence-based principles for health-promoting communication.

*At first, I need to find out how the person lives their life. If I don't know how they eat, what is important to them, and what they themselves want to change, then the whole conversation falls flat*. (IW8)

### 3.3 Take one step at a time

The specialist nurses explained how they follow a step-by-step change principle, focusing on supporting patients to take one small step at a time toward adopting more sustainable lifestyle habits. A key theme was working together with the patient to set small, achievable goals, which helped increase feelings of manageability and long-term sustainability.

*The goals must be ones that the patient themselves feels they can achieve. For us as nurses, they may seem like small steps, but for the patient, they can be huge*. (IW7)

The specialist nurses highlighted the importance of letting patients decide which behaviour they wanted to change, and of establishing mutual agreements through dialogue rather than through templates or guided instructions. They underlined the need to avoid pushing their own agenda and instead create space for the patient's priorities, aiming to strengthen autonomy and commitment to change. Responsibility for making changes was presented as placed mainly on the patients, which the specialist nurses believed fostered empowerment and motivation. Additionally, they pointed out that patients’ existing good habits often served as a valuable starting point, boosting self-confidence and building on current resources in the process of lifestyle change.

### 3.4 Apply understanding and caring

The specialist nurses described how a key part of their work was to use an approach characterized by empathy for the patient's entire life situation. They stressed the importance of trying to identify the underlying causes of the patient's difficulties in following recommendations, as such insights were seen as necessary for creating relevant and realistic strategies for behavioural change. In cases where completely healthy choices were not possible, the specialist nurses worked with the patient to find less unhealthy alternatives, meaning the ‘least bad’ option in a given situation. At the same time, they considered it important to provide clear yet respectful information about the medical risks linked to type 2 diabetes and lifestyle factors. Despite this, the specialist nurses highlighted the importance of accepting the patient's own decisions, even when these conflicted with the advice given, and of maintaining a non-judgmental attitude to foster ongoing dialogue and enable future change.

*Sometimes you have to accept that my own idea of perfection isn’t achievable. The nurse's role is then to find the least bad option that actually works in the patient's daily life*. (IW4)

### 3.5 Taking gender and cultural aspects into account

The specialist nurses accounted for gender-specific elements, multicultural dimensions, and the family and social situation, which they believed influenced the advice.

#### 3.5.1 Gender specific elements

The specialist nurses described how gender-specific factors sometimes influenced conversations about lifestyle habits and self-care. Several pointed out that women generally have lower energy requirements than men, which sometimes created challenges when advising on portion sizes and energy intake.

*I find that sometimes it's harder to get men to eat more vegetables. Women are often more open to this advice, although that's not true for everyone*. (IW6)

Specialist nurses also stated that, in some cases, they found it more challenging to motivate men to increase their vegetable intake, and that women often took on greater responsibility for both shopping and cooking in the family. They emphasized that these patterns were not universal but recurred in meetings with patients.

#### 3.5.2 Multicultural dimensions

The specialist nurses explained that working in a multicultural environment often presented unique challenges related to communication and dietary advice. Several highlighted the risk of communication issues, especially during meetings where an interpreter was needed, and highlighted the importance of ensuring both information and the patient's own words were correctly understood.

*When an interpreter is present, I have to be extra attentive. It's not always guaranteed that everything I say or the patient says will be accurately conveyed, and that impacts the entire situation*. (IW3)

To avoid making assumptions about dietary habits, specialist nurses emphasized that they always start the conversation by asking what the patient currently eats. They noted that significant cultural differences in food traditions and choices can make it hard to offer relevant suggestions without a good understanding of the patient's food and meal culture. In these cases, they tried to begin with the patient's existing eating habits and provide advice based on those, rather than proposing an entirely new diet. The specialist nurses also highlighted that diet is a core part of identity and culture for many patients, such as new arrivals and refugees, meaning that dietary advice must be delivered with great cultural sensitivity and respect for each person's background.

*Food is part of identity, especially for new arrivals and refugees, so advice must be given with cultural respect*. (IW9)

#### 3.5.3 Family and social situation

They emphasized that the patient's family and social environment often play a key role in the ability to implement and sustain lifestyle changes. Several described how a supportive family system can facilitate both motivation and practical steps, while emphasizing that decisions about change must ultimately be made by the patient themselves to ensure sustainability. Socioeconomic conditions were highlighted as a significant challenge, with financial vulnerability limiting access to healthy food and the ability to prioritize self-care.

*Financial constraints have a bigger impact than many realize. If you're barely making ends meet, it can be hard to buy fresh vegetables or prioritize your health, even if you want to*. (IW6)

The specialist nurses expressed that more effort should be made to reach socially vulnerable groups with targeted interventions. They also described patient organizations as an essential complement to healthcare, especially since they can offer knowledge, community, and continuity. Furthermore, the value of promoting participation in various peer support groups was highlighted, as these were seen as contributing to increased motivation, normalization of challenges, and a sense of shared experience among people with type 2 diabetes.

*Patient associations and various support groups are an important complement. They provide both knowledge and community, and many say that this is where they find the motivation to continue*. (IW10)

### 3.6 Challenges associated with implementing changes to prevent type 2 diabetes

The specialist nurses highlighted patients’ lack of motivation, along with their difficulties in implementing new habits in daily life, and the clinical time constraints as obstacles to reaching patients with the healthy message.

#### 3.6.1 Patient lack of motivation

Specialist nurses explained that patients' lack of motivation when only being at risk sometimes hindered work on lifestyle changes, as they did not yet see themselves as ill. They expressed that the willingness to change generally increased first with an acute diagnosis or when complications arose. They also observed that some patients were mainly driven by external factors, such as physical appearance or social status, rather than health concerns. Adherence to recommendations was often described as low, particularly when the advice was seen as restrictive or limited their ability to eat as usual. Several specialist nurses said that patients sometimes viewed dietary advice as a ‘narrowing of the eating window,’ which could cause resistance and a sense of lost freedom. These perceptions highlighted a recurring challenge in balancing medical advice with patients' quality of life and autonomy.

*Many at-risk individuals don't see themselves as ill, so their motivation to change remains low. Often, the desire to change only appears after an acute diagnosis or when complications develop. Otherwise, they view the advice as a restriction, as if their ‘eating window’ is shrinking, which can easily lead to resistance*. (IW5)

#### 3.6.2 Implementation difficulties in daily life

The specialist nurses identified several barriers that hindered the implementation of dietary and lifestyle recommendations in clinical practice. They described that younger patients often relied more on information from the internet, which sometimes led to mistrust of professional advice and made it harder to develop shared strategies.

*For some, it can be difficult to apply this advice in everyday life, especially when patients search for a lot of information online and sometimes trust it more than they trust us*. (IW2)

They also believed that there was a risk that their preconceived opinions could lead to dietary advice that is not sufficiently personalized, such as assuming that individuals of different ethnicities have not adapted their diets to Swedish habits. Some patients were described as particularly challenging to reach, especially when lifestyle changes felt burdensome or did not align with their daily lives. Specialist nurses also observed that women sometimes faced practical obstacles at home, as dietary changes were not always accepted by other family members, which could require preparing several different dishes.

#### 3.6.3 Clinical time constraints

A recurring challenge that specialist nurses mentioned regarding clinical work was the limited time available per patient visit. They felt that the designated consultation time was often not enough to cover both medical follow-ups and in-depth lifestyle discussions. The time shortage also made it challenging to prepare for each meeting, especially when the patient's situation was complex or needed tailored advice. This was seen as impacting both the quality of discussions and the ability to provide long-term, person-centred care. Several specialist nurses noted that the patient group of prediabetes and type 2 diabetes tends to be a low-priority patient group within the healthcare organization.

*…and then, when it is a patient with a complex situation, it becomes almost impossible to provide person-centred advice, and unfortunately, this particular group with prediabetes or T2D is often given the lowest priority in the organization*. (IW11)

### 3.7 Requesting additional support and resources

The specialist nurses requested better cultural competence education and easier access to new knowledge. An important aspect to fulfill their task was also organizational backing.

#### 3.7.1 Cultural competence education

They expressed a strong need for more training in cultural competence, especially regarding the eating habits and dietary norms of different cultures. They felt that dietary counselling often required understanding of specific food cultures to provide relevant, personalized recommendations, and several described such training as ‘really good and interesting.’ The specialist nurses stated that guidelines considering gender-specific and ethnic factors could help make counselling more effective, as it felt more ‘right’ to give advice perceived as culturally and socially appropriate.

*In order to give relevant dietary advice, I need to understand their food traditions. General knowledge alone isn't enough. I must know how their specific culture views food, or else the advice risks being inaccurate. Training in cultural competence would really help us give advice that feels right for the patient*. (IW3)

At the same time, they emphasized the importance of avoiding assumptions about patients' eating habits and always starting by exploring each individual's actual dietary patterns. For example, when an Asian woman was married to a Swedish man, it was not certain that they would cook Asian food. Additionally, they pointed out that not only education, but also enough consultation time and opportunities for follow-up would be necessary to implement culturally adapted advice successfully.

#### 3.7.2 Easy access to new knowledge

The specialist nurses expressed a need for more accessible and up-to-date knowledge to share with patients, particularly regarding lifestyle and health. They requested updated, evidence-based information material online, as existing brochures were often perceived as outdated. They also requested that dietary advice be made more accessible, for example, through written plans, preferably translated into the patient's native language, in order to support understanding and participation.

*The material we have is often outdated. We need easily accessible, current advice – preferably online and available in multiple languages, so that both we and our patients can receive the correct information immediately. That would significantly improve the advice we provide*. (IW5)

The specialist nurses described how teaching patients about meal plans and other concrete advice, such as exercise after carbohydrate-rich meals, was of great educational importance to patients. Several emphasised the benefits of group-based initiatives where patients could learn and reflect together, reduce guilt and share experiences.

*Patients need clear and practical knowledge, such as specific meal plans or advice on taking a walk after a carbohydrate-heavy meal. I also strongly believe in group-based initiatives, like cooking groups or digital programs, where people can learn from each other and receive support*.(IW4)

Suggestions included cooking groups where participants could try new dishes, discuss the effects of diet and receive direct feedback. Specialist nurses also expressed that digital group programmes could be a way to reach more people and lower the threshold for participation. Another need that was identified was to develop knowledge banks or libraries with information about different food cultures and foods to enable more personalised advice. Digital dietary guidelines were also requested, both as support in clinical decisions and to offer patients more relevant and situation-specific guidance.

#### 3.7.3 Organisational backing

The specialist nurses emphasised that stronger organisational support would be crucial to improving preventive work.

*We need clearer organizational support. There should be procedures in place to identify high-risk patients earlier and opportunities to offer more structured dietary consultations at an earlier stage. As it stands now, much depends on the individuals*. (IW9)

They called for clearer procedures for early identification of patients at risk of type 2 diabetes and easier opportunities to offer structured dietary advice. Several criticised outdated guidelines, arguing that updated, evidence-based recommendations are needed to improve the quality of care. Regular follow-ups were described as key to strengthening compliance and highlighting changes, with biomarkers such as HbA1c and plasma glucose often used as motivational tools to concretise treatment effects. At the same time, they emphasized the importance of avoiding quick fixes and instead working towards long-term, sustainable change.

## 4 Discussion

The specialist nurses described challenges in providing tailored dietary advice to patients at risk of type 2 diabetes, taking into account their whole life situation, such as social, cultural and gender aspects. As described in the results, the specialist nurses found it a complex task, as they had to adapt to patients' diverse life situations without additional education or encouragement from managers in the organization. They focused on patients' goals and strategies with an effort to build trust, listen actively, and personalize advice to each patient’s situation, which was seen as key to support meaningful lifestyle changes. This matches previous research indicating that personalized counselling increases patient engagement and promotes sustainable self-management routines (Cheng et al., 2025). Patients’ preference for clear, tailored advice and explanations about health risks, as noted in earlier studies (Arnardóttir et al., 2024; Graue et al., 2023), aligns with the specialist nurses’ focus on motivational interviewing and non-judgmental communication. Together, these viewpoints highlight the importance of communication strategies that foster autonomy and empower patients in their decision-making (Linnet Olesen and Jørgensen, 2023; Lönnberg et al., 2019, 2020). They gave a detailed description of how they used “one step at a time” strategies. By supporting patients to set small, achievable goals, specialist nurses foster a sense of manageability that can reduce the overwhelm often linked to major lifestyle changes. This step-by-step approach aligns with recommendations for behavioural change interventions, which emphasize that gradual progress improves sustainability and self-efficacy (Dunkel et al., 2025). Similar patterns have been seen in diabetes prevention studies, where small, patient-defined changes support long-term adherence (Schmidt et al., 2023). By applying understanding and caring, they also highlighted the emotional and contextual aspects of lifestyle counselling. The specialist nurses emphasized empathy and a comprehensive understanding of patients’ lives, carefully examining underlying causes of difficulties after providing recommendations. This aligns with research showing that lifestyle behaviours are rooted in broader social, cultural, and psychological contexts (Hao et al., 2025; Hood et al., 2015). By accepting patients’ choices, even when they do not align with medical advice, and assisting them in identifying “least bad” options, specialist nurses maintain a therapeutic relationship that encourages ongoing communication (Wu et al., 2024). This relational continuity is known to enhance readiness for change and improve patients’ long-term engagement in care (Lönnberg et al., 2019, 2020).

The specialist nurses were accounting for several aspects that were influencing their advice. An important aspect revealed in this study involves how specialist nurses address gender-specific and multicultural considerations. Their perceptions with gender patterns, such as women’s lower energy needs or men’s reduced vegetable intake, mirror findings from earlier research indicating that lifestyle counselling often intersects with social norms and household roles, as supported by recent evidence showing that sociocultural expectations and household dynamics influence lifestyle behaviours (Visagie et al., 2024). Likewise, the difficulties in offering culturally tailored dietary advice are well documented. Specialist nurses’ approach of starting with patients’ existing food habits aligns with best practices in culturally sensitive counselling, which emphasize avoiding assumptions and grounding recommendations in the patient's own cultural and culinary background (Lönnberg et al., 2022; Saenz et al., 2025). This approach is especially relevant in diverse populations and among individuals with migrant backgrounds, where food practices are deeply connected to identity, a connection extensively described in recent literature on culturally adapted diabetes care (Hao et al., 2025). Family dynamics and social circumstances also emerged as influential factors. The specialist nurses’ descriptions of family support as both a facilitator and a barrier mirror previous qualitative findings that lifestyle changes often depend on collective household practices, as demonstrated by recent research highlighting how family routines shape self-management, shared norms, and emotional climate (Visagie et al., 2024). Their observations regarding socioeconomic barriers, including financial insecurity limiting access to healthy foods, illustrate well-established inequalities in diabetes risk and prevention, consistent with evidence showing that social determinants of health, such as income, food access, and living conditions, significantly shape diabetes outcomes and behaviour change capacity (Hao et al., 2025; Waage et al., 2025). These insights underscore the need for public health initiatives that address structural determinants of health, extending beyond individual-level counselling, a point reinforced by lifestyle counselling research that emphasizes the necessity of structural and system-level support to enable sustainable behaviour change (Lönnberg et al., 2022).

However, they were also facing several challenges in patient meetings and in the outcomes of their advice, as people at increased risk of type 2 diabetes often do not identify with a patient role and may therefore question the relevance and urgency of lifestyle advice. As a result, specialist nurses are required to navigate counselling situations where the goal is to promote change in the absence of explicit illness, symptoms, or shared perceptions of risk. This aligns with known issues in prediabetes care, where asymptomatic individuals may not feel an immediate need to change, as shown in recent qualitative studies indicating that people with early-stage or asymptomatic diabetes often lack urgency for lifestyle adjustments (Visagie et al., 2024). The specialist nurses’ descriptions of external motivators, such as appearance, reflect the complex interaction among health, identity, and social pressures, which aligns with research showing that emotional, identity-related, and social factors strongly influence readiness for behaviour change in diabetes (Hao et al., 2025). Their difficulty in balancing lifestyle advice with patients’ quality of life underscores the delicate nature of behavioural counselling, a challenge highlighted by current lifestyle counselling research that emphasizes the need to balance medical goals with patients’ everyday realities and well-being (Lönnberg et al., 2022). Organizational constraints were another prominent barrier. The specialist nurses reported limited time for clinical consultation, competing clinical priorities, and a lack of emphasis on preventive efforts. These issues echo earlier findings that they often lack the structural conditions needed to provide comprehensive lifestyle counselling, a problem supported by recent research showing that organizational workload, limited resources, and time constraints hinder their ability to offer person-centred lifestyle support (Pot et al., 2025). Limited time not only reduces the depth of discussions but also disrupts follow-up continuity, which is vital for effective behaviour change. Studies demonstrate that consistent follow-up and relational continuity are crucial for maintaining motivation and behavioural adherence (Hao et al., 2025; Lönnberg et al., 2022; Unnikrishnan et al., 2025). These structural challenges highlight the need for organizational reforms, including longer consultation periods, clearer risk identification procedures, and greater access to updated, evidence-based guidelines. This aligns with calls in current diabetes care literature for system-level change and structural support to enhance preventive efforts (Hao et al., 2025).

Lastly, the specialist nurses requested additional support and resources, particularly for improved cultural competence training, easy access to up-to-date educational materials, and digital tools, to further demonstrate their desire to modernize practice and better customize interventions for diverse patient groups. Their interest in group-based formats, such as cooking classes or digital group programs, aligns with evidence indicating that peer support and experiential learning enhance motivation and normalize challenges (Echouffo-Tcheugui et al., 2025, 2018). To further support this argument, it is also important to consider the technological literacy of the target patient groups. Several specialist nurses observed that younger patients, in particular, tend to trust online information more than professional advice, which influences how digital tools and educational resources should be designed and implemented.

Recent studies show that peer-supported, culturally sensitive group formats improve engagement and self-management in diabetes care (Saenz et al., 2025). Additionally, the call for knowledge banks focused on food cultures underscores the complexity of dietary counselling in multicultural settings and the need for accessible, practical resources. This need is strongly emphasized in recent research on culturally sensitive diabetes management, which highlights how food practices are deeply connected to identity, culture, and social belonging (Hao et al., 2025; Lönnberg et al., 2022).

### 4.1 Limitations

This study has some limitations that should be acknowledged when interpreting the findings. First, the sample size was relatively small, which is common in qualitative research but may limit the range of perspectives captured. Second, the sample was predominantly female. Although this reflects the actual gender distribution within the professional groups studied, diabetes specialist nurses and district nurses in Swedish primary health care, it nonetheless limits the representation of male perspectives. Future studies may benefit from targeted efforts to include a more gender-diverse sample to explore potential differences in views and experiences. Third, participants were recruited through snowball sampling. While this method can be beneficial in qualitative research, particularly because participants often know colleagues who are knowledgeable and willing to contribute, it also risks creating a more homogeneous sample. Snowball sampling may result in including participants who share similar opinions or professional values, thus reducing diversity in perspectives. However, given the exploratory nature of the study, this method was deemed appropriate for accessing information-rich participants with valuable insights into the topic.

Additionally, all participants were recruited from northern Sweden, which might limit how well the findings apply to other geographic and clinical settings. The region is relatively homogeneous in terms of population characteristics and may differ from urban or more multicultural areas where healthcare interactions are influenced by greater ethnic diversity and more complex socio-cultural factors. Moreover, organizational conditions in northern Sweden, such as resource availability and care structures, may not fully represent those in larger urban healthcare environments. These contextual factors should be kept in mind when considering the relevance of the findings to other settings.

As with most qualitative research, it is up to the reader to determine the relevance of the results to other settings. To enhance transferability, we have provided detailed descriptions of the study context, participant characteristics, and data collection procedures. A notable strength of the study is the composition of the international research team. The researchers come from different countries, with diverse academic backgrounds and professional perceptions, which enriched the analytical process and reduced the risk of individual interpretative bias. The analysis further benefited from the group’s combined expertise in qualitative methods and diabetes care, enhancing both the credibility and depth of the interpretations. Overall, while the study’s methodological choices introduce certain limitations, they also reflect the practical realities of qualitative inquiry. By providing transparency in sampling, analysis, and contextual descriptions, we aim to help readers evaluate the trustworthiness and applicability of the findings.

### 4.2 Implications for practice

Enhancing preventive diabetes care requires organizational commitment to improving the conditions under which specialist nurses work. Providing adequate consultation time, establishing clear routines for early identification of at-risk patients, and ensuring access to current, evidence-based guidelines would create a more stable foundation for effective counselling. Ongoing training in cultural competence is essential since specialist nurses often encounter diverse food traditions, migration backgrounds, and gender-related self-care patterns that influence patients’ ability to adopt lifestyle changes. They specifically requested more structured cultural competence training, such as access to ‘knowledge banks’ compiling multicultural food traditions to better tailor nutritional guidance. They emphasized that understanding concrete, culture-bound eating patterns is necessary to provide advice that feels relevant and socially appropriate for each patient. Lastly, the specialist nurses requested additional support and resources, particularly improved cultural competence training, easy access to current educational materials, and digital tools, demonstrating their desire to modernize practice and better personalize interventions for diverse patient groups. Their interest in group-based formats, like cooking classes or digital group programs, aligns with evidence showing that peer support and experiential learning boost motivation and normalize challenges. To further enhance this, it may also be useful to consider the technological literacy of the target patient groups, as several specialist nurses noted that younger patients tend to trust internet-based information quite a bit, sometimes even more than professional advice, with implications for the design and implementation of digital tools and educational support.

To promote person-centered care, healthcare organizations should offer easily accessible, up-to-date educational materials, both digital and printed, along with translated resources as needed. Expanding digital and group-based interventions, including peer-support models, can supplement individual counselling and increase outreach. Collectively, these strategies can improve the relevance, feasibility, and sustainability of dietary advice and better support patients in making meaningful lifestyle changes.

## 5 Conclusions

This study shows that specialist nurses are committed to helping patients at risk of type 2 diabetes adopt healthier eating habits, yet their efforts are shaped, and often constrained, by structural, cultural, and motivational challenges. They used person-centred strategies, empathy, and individualized goal-setting, but their ability to influence change was limited by patients' lack of motivation and gender, complex life situations, and restricted consultation time. They also highlighted the need for improved cultural competence, accessible and updated educational resources, and stronger organizational support. In conclusion, the findings reinforce the need for both individualized behavioural support and broader system-level changes to effectively prevent diabetes. Strengthening organizational frameworks, expanding access to culturally adapted materials, and promoting collaborative care models—including digital and in-person formats may significantly enhance the preventive impact of specialist nurses and support patients in making sustainable lifestyle changes (Zhang et al., 2025).

## Declaration of generative AI and AI-assisted technologies in the manuscript preparation process

During the preparation of this work, the authors used Copilot in order to support language refinement and improve clarity of academic writing. After using this tool, the author(s) reviewed and edited the content as needed and take full responsibility for the content of the published article.

## Funding

This project is supported under the framework of the European Research Area Personalized Medicine Joint Transnational Call (ERA-PerMed JTC2022), by the South African Medical Research Council (SAMRC) with funds received from the Department of Science and Innovation, Bundesministerium für Bildung und Forschung (BMBF), Germany; Vinnova (Dnr: 2022-00547) and the Swedish Research Council (Vetenskapsrådet; Dnr: 2022-00924), Sweden.

## CRediT authorship contribution statement

**Åsa Carlsund:** Writing – review & editing, Writing – original draft, Project administration, Methodology, Formal analysis, Data curation. **Åsa Hörnsten:** Writing – review & editing, Resources, Investigation, Formal analysis, Data curation, Conceptualization. **Fatima Hoosen:** Writing – review & editing, Methodology. **Stephen Amoah:** Writing – review & editing, Methodology. **Julia Otten:** Writing – review & editing, Methodology, Data curation.

## Declaration of competing interest

The authors declare the following financial interests/personal relationships which may be considered as potential competing interests:

Carlsund, Hornsten, Hoosen, Amoah, Otten reports financial support was provided by European Research Area. Carlsund, Hornsten, Hoosen, Amoah, Otten reports financial support was provided by South African Medical Research Council. Carlsund, Hornsten, Hoosen, Amoah, Otten reports financial support was provided by Department of Science and Innovation, Bundesministerium für Bildung und Forschung. Carlsund, Hornsten, Hoosen, Amoah, Otten reports was provided by Swedish Research Council. If there are other authors, they declare that they have no known competing financial interests or personal relationships that could have appeared to influence the work reported in this paper.

## Appendix Supplementary materials

Image, application 1

Image, application 2
